# GPCR homo-oligomerization

**DOI:** 10.1016/j.ceb.2018.10.007

**Published:** 2019-04

**Authors:** Graeme Milligan, Richard J Ward, Sara Marsango

**Affiliations:** Centre for Translational Pharmacology, Institute of Molecular, Cell and Systems Biology, College of Medical, Veterinary and Life Sciences, University of Glasgow, Glasgow G12 8QQ, Scotland, United Kingdom

## Abstract

G protein-coupled receptors (GPCRs) are an extensive class of trans-plasma membrane proteins that function to regulate a wide range of physiological functions. Despite a general perception that GPCRs exist as monomers an extensive literature has examined whether GPCRs can also form dimers and even higher-order oligomers, and if such organization influences various aspects of GPCR function, including cellular trafficking, ligand binding, G protein coupling and signalling. Here we focus on recent studies that employ approaches ranging from computational methods to single molecule tracking and both quantal brightness and fluorescence fluctuation measurements to assess the organization, stability and potential functional significance of dimers and oligomers within the class A, rhodopsin-like GPCR family.

## Graphical abstract

Cartoon depictions of **(a)** Dynamic interchange between receptor monomers, (homo, orange GPCR1, and hetero, orange GPCR1 and green GPCR2) dimers and oligomers which may be ligand regulated (orange sphere). **(b)** Signalling cross-talk between protomers of a dimer. Left: ligand (orange sphere) binds to one protomer and the conformational change transmitted to the second protomer results in signalling through activation of a Gα subunit (blue). Right: ligand (green sphere) binding to the second protomer may modulate signalling (large or small yellow flash).

**Figure:**
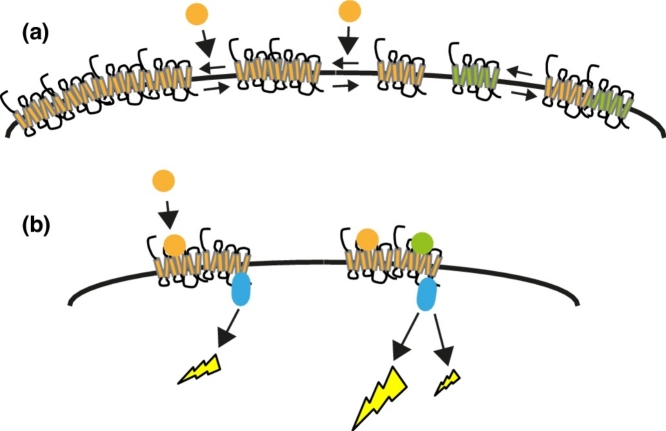

**Current Opinion in Cell Biology** 2019, **57**:40–47

This review comes from a themed issue on **Cell signalling**

Edited by **Wouter H Moolenaar** and **Tamás Balla**

For a complete overview see the Issue and the Editorial

Available online 16th November 2018

**https://doi.org/10.1016/j.ceb.2018.10.007**

0955-0674/© 2018 The Authors. Published by Elsevier Ltd. This is an open access article under the CC BY license (http://creativecommons.org/licenses/by/4.0/).

## Introduction

The human genome encodes more than 800 seven transmembrane-domain, G protein-coupled receptors (GPCRs). In recent years enormous advances in structural information on GPCRs have emerged in parallel with methods to stabilize these proteins when they are extracted from cellular membranes. Despite this, one area that has remained uncertain is the quaternary organization of these proteins in their native environment [1, 2]. The numerically predominant rhodopsin-like, or Class A, GPCRs are generally described as monomers. However, evidence emerging from a broad range of approaches has shown that they can form both dimers and higher-order oligomers with protomers of either the same receptor (homo-dimers/oligomers) or with partners of the same sub family and even with GPCRs which respond to different ligands (hetero-dimers/oligomers).

## Homo-oligomerization of class A GPCRs

There is still significant debate about the functional significance, the molecular basis of, the extent, and even the existence [3], of dimers and oligomers of class A GPCRs. Reasons for this are complex but include that many studies have been performed without adequate controls, that many have been limited to experiments performed in simple transfected cell systems, and that there are a wide range of Class A GPCRs which, although all possessing the same general architecture of seven linked transmembrane domains, may not all be defined by a single pattern of structural organization or with equivalent self-avidity. Moreover, many reported studies are largely qualitative and have failed to address the proportion of a receptor that might be present as dimers or oligomers at steady-state and how this might be regulated. There is also a potential issue that different techniques are not equally well suited to analysis of trans-membrane protein interactions at different levels of expression. In physiological settings some GPCRs may be expressed at levels of only a few hundred copies per cell, whilst in the central nervous system some receptors are expressed at hundreds of times this level. Covalent interactions are not generally involved in maintaining quaternary structure of Class A receptors. It must be expected, therefore, that both expression levels and intrinsic affinity will potentially determine the extent of interactions via mass-action [4, 5]. Comparisons of outcomes for a single GPCR can illustrate the variety of outcomes and opinions. When observed via total internal reflection fluorescence microscopy the muscarinic acetylcholine M_2_ receptor (M_2_R) appeared to exist predominantly as a monomer but was able to reversibly form dimers at the plasma membrane of each of transformed Chinese Hamster Ovary cells, a cardiac cell line, primary cardiomyocytes and tissue slices from pre-natal and post-natal mice [6]. By contrast, fluorescence correlation spectroscopy (FCS) with photon counting histogram studies reported this receptor as being organized intrinsically as a dimer in a transfected cell line [7], whilst Fluorescence Resonance Energy Transfer (FRET)-based approaches, including the application of step-wise photo-bleaching protocols, and ligand binding studies have been consistent with and interpreted as showing this receptor to exist predominantly as tetramers in both transfected cells and native tissue [8,9^•^]. It is challenging to bring consensus to such varying reports. A very recent study in which the oligomerization characteristics of three distinct class A GPCRs, the β_2_-adrenoceptor (β_2_-AR), the cannabinoid CB_1_ receptor and opsin, were investigated in proteoliposomes highlighted how the extent of receptor organization is receptor type specific and sensitive to environmental effectors, including protein density and membrane curvature [10^••^], potentially explaining some of the disparate reports present in the literature. Although only one subset of many approaches that have been employed [11] methods based on Resonance Energy Transfer (RET) techniques have been central to the development of studies on Class A receptor quaternary organization. Importantly, a number of recent studies have brought the rigor of mathematics and physics to the interpretation [10^••^,^12^,^13^] but studies are still difficult to transfer from either *in vitro* purified protein and reconstitution studies or the use of transfected cell systems into more native cells and tissues. Recent times have seen a broader palette of approaches emerge, including single fluorescent molecule tracking studies [14,15] and analysis of quantal brightness linked to fluorescence fluctuations of fluorophore-tagged GPCRs [4,16].

## Many GPCR homomeric complexes are transient

Class A GPCR dimers may be transient species, at least when present at modest levels [17,18,19^•^] and have shown rapid and dynamic interactions with half-times in the second to sub second scale [19^•^]. Single-molecule analysis of fluorescently labelled β_1_-adrenoceptors and β_2_-adrenoceptors indicated these were both organized as a mixture of different sized complexes at the plasma membrane of Chinese Hamster Ovary cells, with the β_2_-AR showing higher complexity at equal expression levels [17]. Moreover, the complexity of particularly the β_2_-AR was observed to increase with receptor density at the cell surface, consistent both with transient interactions and effects of mass-action [17]. Further evidence for transient interactions between individual protomers being driven by mass-action has accumulated. Increasing quaternary complexity with higher levels of expression has also been reported via single molecule tracking of the dopamine D_2_ receptor (D_2_R) [18,19^•^]. A key question in many studies has been whether the half-life of the interaction between receptor protomers is modulated by the binding of ligands to the receptor. For example, the half-life of the interaction between unliganded D_2_Rs was measured to be about 0.5 s at 24 °C and although binding of antagonists to the receptor did not alter the observed equilibrium between monomers and dimers, the binding of agonists did [18]. Similarly, others have measured the half-life of the interaction between unliganded-D_2_R protomers to be roughly 68 ms at 37 °C and, although the addition of the antagonist UH-232 did not alter this, treatment with either the endogenous agonist dopamine or the synthetic agonist quinpirole stabilized dimers, increasing the half-life to 99 and 104 ms, respectively [19^•^]. Although effects of ligands on GPCR dimerization remains a contentious issue it is clearly central to fully understand the potential significance of receptor dimers and oligomers.

## Outcomes from fluorescence fluctuation studies

**Figure 1:**
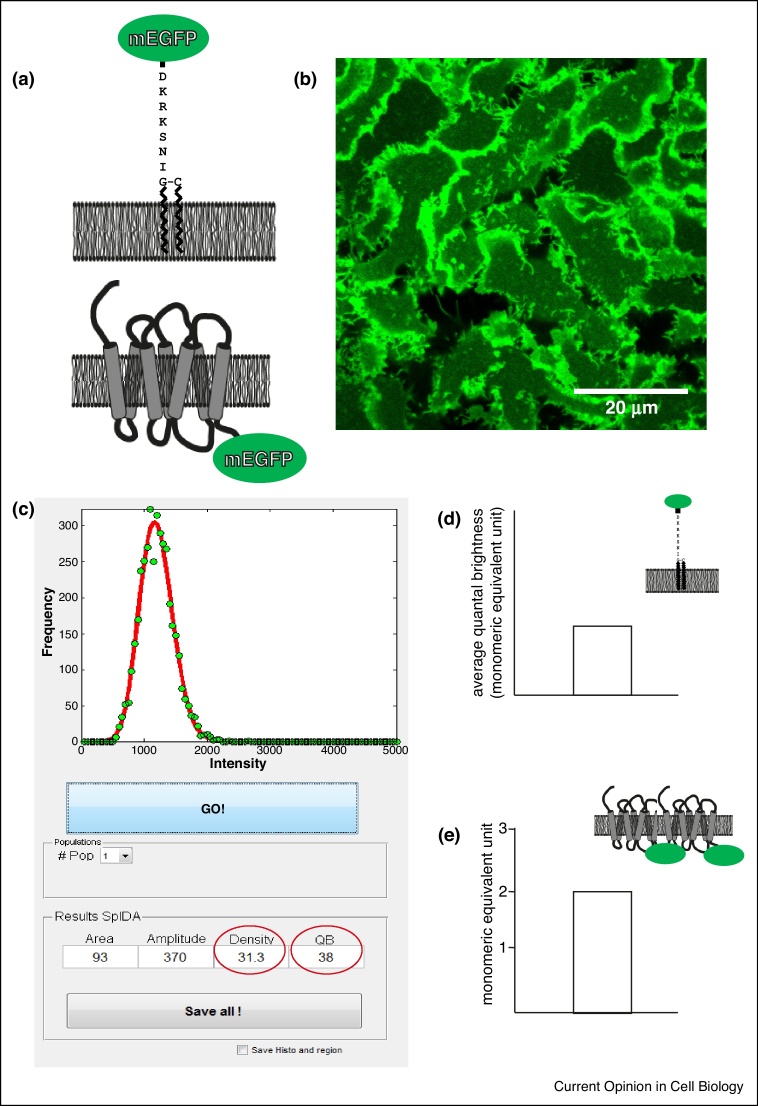
Determination of receptor oligomeric structure by Spatial Intensity Distribution Analysis (SpIDA). **(a)** Constructs for determination of monomeric quantal brightness: upper panel, monomeric enhanced green fluorescent protein (mEGFP) is linked to the plasma membrane via a myristoylation/palmitoylation motif: lower panel A GPCR tagged at the intracellular C-terminal tail with mEGFP. **(b)** Constructs are expressed in, for example Flp-In T-REx-293 cells, and confocal microscope images obtained. **(c)** Images are opened in the SpIDA software (https://neurophotonics.ca/software), regions of interest selected and analysed for protein density and quantal brightness. **(d)** Average quantal brightness for mEGFP is determined using the myristoylation/palmitoylation-linked construct shown in (a). This generates a value of monomeric equivalent unit. **(e)** Measurements of the labelled GPCR yield an average quantal brightness value which can then be compared to the monomeric quantal brightness to determine oligomeric organization. See [4,16] for further details.

**Figure 2:**
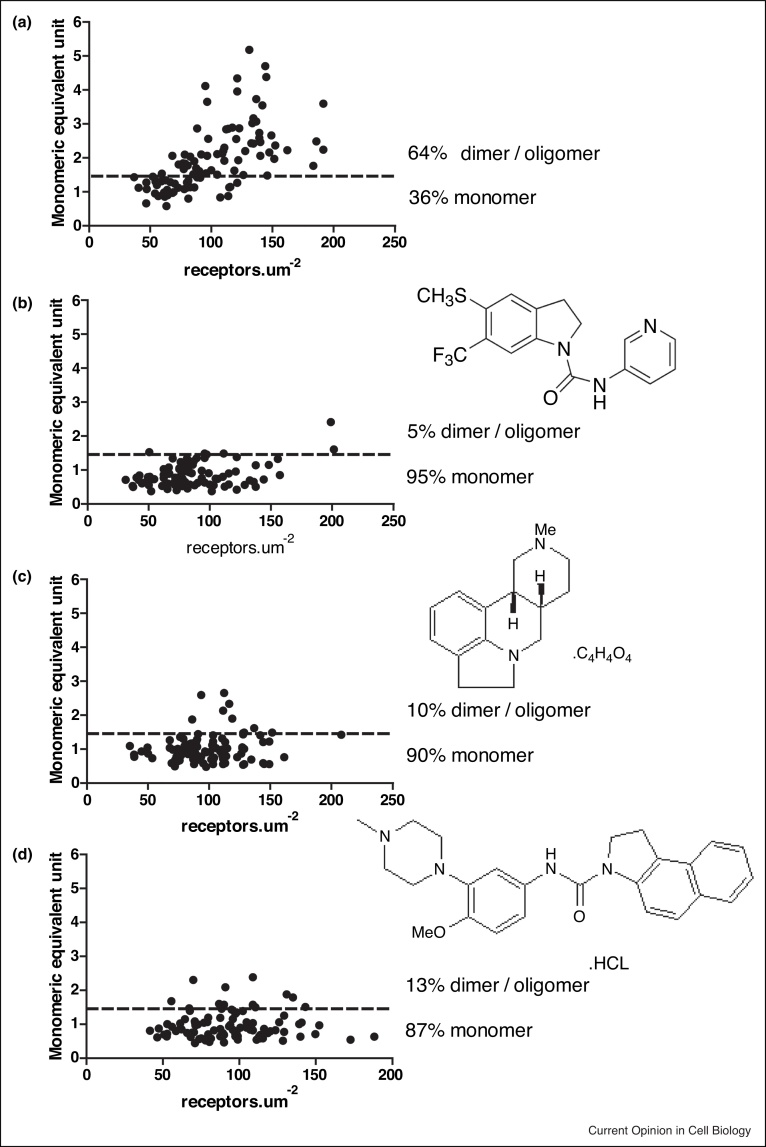
Effect of antagonist treatment on the serotonin 5-HT_2C_ receptor quaternary organization. SpIDA showing measures of individual regions of interest plotted as receptor number (density.μm^2^) versus monomeric equivalent units in Flp-In T-REx-293 cells expressing 5-HT_2C_-mEGFP [14,20]. **(a)** untreated, (**b)**, **(c)** and **(d)** Cells treated with SB-221284 (2,3-dihydro-5-(methylthio)-N-3-pyridinyl-6-(trifluoromethyl)-1H-indole-1-carboxamide) (75 nM), SDZ SER 082 fumarate ((+)-cis-4,5,7a,8,9,10,11,11a-octahydro-7H-10-methylindolo[1,7-bc][2,6]-naphthyridine fumarate) (5 μM) or S32212 hydrochloride (*N*-[4-methoxy-3-(4-methylpiperazin-1-yl)phenyl]-1,2-dihydro-3-*H*-benzo[*e*]indole-3-carboxamide) (1 μM) respectively for 24 hours. Chemical structures for each ligand are shown. Each of these ligands has antagonist activity at the 5-HT_2C_R and was used at a concentration calculated to be 10 × K_i_. In each case treatment with the ligand results in predominantly monomeric status of the receptor

Studies based on quantal brightness and fluorescence fluctuation analysis have also provided support for ligand regulation of receptor organization. The most widely used of these methods has been Spatial Intensity Distribution Analysis (SpIDA) [4]. Imaging of the basolateral membrane of mammalian cells expressing a GPCR of interest tagged at the intracellular carboxyl-terminal tail with a monomeric version of a fluorescent protein such as enhanced green fluorescent protein (mEGFP) has been used to explore both basal organizational state of various Class A GPCRs and the effect on this of therapeutically relevant medicines (Figure 1). In studies on the serotonin 5-HT_2C_ receptor (5-HT_2C_R), which is a target for anti-obesity medications, at steady-state and at relatively low density the receptor was predominantly monomeric, but with a clearly observed fractions of dimers [20]. However, with increasing receptor density there was a strong correlation with increasing receptor organizational complexity, and a substantial proportion of higher-order oligomers as well as dimers was observed [20], demonstrating that the quaternary organization of class A GPCRs is not restricted to dimerization. A number a distinct ligand chemotypes with affinity as 5-HT_2C_R antagonists acted to decrease the complexity of organization of this receptor, essentially converting the receptor to monomeric state [20]. Ongoing studies have verified and extended these initial findings (Figure 2), including for the drug ritanserin, which was the ligand used to stabilize the 5-HT_2C_R for crystallization studies [21]. This suggests that all 5-HT_2C_R antagonists may destabilize dimers and oligomers of this receptor. As might be anticipated for pharmacological ligands that bind reversibly to the target, the effect of these compounds was concentration-dependent and following their wash-out the initial complexity of receptor organization was fully restored [20]. Interestingly, and in contrast to the lack of effect of certain D_2_R receptor antagonists reported by [18], in SpIDA studies on the closely related dopamine D_3_ receptor (D_3_R) a number of, but not all, antagonists also promoted monomerization of the receptor [22^••^]. As noted earlier, [10^••^] have recently highlighted the effects that features such as membrane curvature and lipid makeup can have on Class A GPCR organization. It is important, therefore, to establish that effects of ligands actually reflect binding of the ligand to the receptor, rather than being produced by an off-target effect on the membrane containing the receptor. Marsango *et al.* [22^••^] assessed this by generating a mutant of the D_3_R that is unable to bind the antagonist spiperone. This mutated receptor showed the same basal quaternary organization as the wild type at equal receptor density but now this was completely unaffected upon addition of spiperone [22^••^]. Initial applications of SpIDA and related techniques highlighted the capacity to assess protein quaternary organization from simple confocal images and that this could be used in native tissues [16]. It is thus likely that these types of techniques will now be used more widely in both native tissues if appropriate fluorophores can be attached to or incorporated into GPCRs of interest, or in tissues isolated from transgenic animals expressing a fluorophore-labelled GPCR.

## Are specific interfaces required for dimerization/oligomerization?

Observations of ligand effects on receptor quaternary organization are inherently interesting but provide little direct insight into mechanism(s). In earlier studies Marsango *et al.* [23] had taken a mutational approach to define potential dimerization interfaces of the D_3_R. This lead to predictions of roles for both sections of transmembrane domain I and for amino acids in transmembrane domains IV and V and, therefore, indicated that there must be multiple interfaces able to promote and stabilize dimeric interactions. Whilst both spiperone and haloperidol promote monomerization of the D_3_R they appear to do so by different mechanisms [22^••^]. Molecular dynamics simulations of the binding of these ligands to the D_3_R indicated that spiperone increased the distance between reference carbon atoms near the extracellular face of transmembrane domains IV and V compared to the apo-protein. By contrast, although binding of haloperidol did not alter this distance it instead increased the distance between reference carbon atoms in transmembrane domains I and II [22^••^]. By contrast ligands such as eticlopride did not alter either of these intra-molecular distances and also did not affect basal receptor quaternary organization [22^••^]. A clear conclusion from these studies is that regions of each of transmembrane domain I and II, and domains IV and/or V, contribute to distinct dimerization interfaces (Table 1). It is likely that similar combinations of molecular dynamics simulations of ligand binding and direct analysis of receptor quaternary organization will provide further insights for other GPCRs.

**Table 1:** Summary of approaches used to determine the molecular mechanism of protomer-protomer interaction.

Technique	GPCR	TMs involved in dimerization	References
X-ray crystallography	Adenosine A_1_	IV-V	[38]
Biochemical approaches Cross-linking Gel filtration Isolated TM addition	CCR5 chemokine Rhodopsin Thromboxane A_2_	V I-II, IV-V I	[39] [40] [30^••^]
Biophysical approaches BRET FRET Fluorescence Correlation Spectroscopy (FCS) Pulsed-Interleaved excitation Fluorescence Cross-Correlation Spectroscopy (FCS-FCCS) FCS Bimolecular Fluorescence Complementation	Rhodopsin Angiotensin II type 1 β2-AR Dopamine D_3_ Muscarinic M_3_ CCR5 chemokine Cone opsins Cone opsins Cone opsins β2-AR β2-AR	I-II, IV-V I-IV, IV-V, VI-VII I-Helix VIII I-II-Helix VIII, IV-V I-II-Helix VIII-V-VI-VII V V V V I-Helix VIII I-Helix VIII	[40] [41] [29] [23] [42] [39] [28] [28] [28] [29] [29]

Not all studies have suggested GPCR quaternary structure to be so dynamic. In studies that examined a number of Class A receptors using FCS and photon counting histograms [7] outcomes were interpreted as favoring fixed dimeric organization across a substantial range of receptor densities. This may, however simply reflect the mean distribution observed using such ensemble-based methods. Moreover, Wells and colleagues have consistently supported a sustained, at least tetrameric, arrangement for the M_2_R at varying receptor densities [8]. Despite these examples it now appears clear that certain receptors have a limited ability to maintain dimeric organization. For example, in essentially all detailed studies the β_1_-adrenoceptor has acted essentially as a monomeric protein. Similarly, SpIDA on HEK 293-derived cells engineered to express a monomeric eGFP-tagged form of the muscarinic M_1_ receptor showed that at steady-state only a small portion of the regions of interest examined on the basolateral membrane of these cells contained the receptor organized predominantly as dimers/oligomers and this was the case across expression densities ranging from 20–120 receptor per μm^2^ [24]. In this example, however, the selective M_1_ receptor antagonists pirenzepine and telenzepine increased, rather than decreased, the level of steady-state association, although even at concentrations of the drugs predicted to provide full receptor occupancy this remained far from quantitative. Moreover, common anti-muscarinic drugs such as atropine did not produce this effect [24]. Recent studies using acceptor photo-bleaching-FRET and computational approaches, including molecular dynamics simulations combined with multi-ensemble Markov state models reached similar conclusions regarding the low proportion of steady-state dimers of the μ-opioid receptor (MOR) [25].

## Dimerization affects tissue function and responsiveness

As highlighted earlier, although their overarching seven transmembrane domain architecture might suggest conserved interaction interfaces, protein interfaces that encourage dimerization are still relatively poorly defined and this area certainly lacks a single unified conclusion [26]. Moreover, as dimer contacts frequently appear to be transient it could be argued that no specific interface would be dominant. Synthetic peptides derived from specific transmembrane domains have been central to addressing this question, and this approach recently translated from *in vitro* to *in vivo* studies that indicated a key role for transmembrane domain I in interactions between rhodopsin protomers [27]. Alternatively, however, a mutagenic approach instead suggested a key role for transmembrane domain V residues in human red cone opsin [28]. Moreover, assuming that a significant fraction of the β_2_-AR can indeed exist as dimers, transmembrane domain I may play an integral role [29]. This is also the case for the thromboxane A_2_ receptor [30^••^] and, intriguingly in the context of physiology and disease, patients with mutations in this region of the receptor that show reduced dimerization in *in vitro* studies have also been shown to suffer bleeding disorders associated with reduced platelet function [30^••^]. This provides a potentially key link between poor dimerization capability and reduced receptor function *in vivo* and, therefore, a patho-physiological significance of Class A GPCR dimerization. Studies on other GPCRs have implicated other transmembrane domains. For the angiotensin AT_1_ receptor, each of domains IV, V, VI, and VII were recently implicated [31]. Clearly such differences in results may reflect different modes of interaction, or indeed that different interfaces are required to construct dimers, trimers, and tetramers, for example [32] as there is now less of a consensus that quaternary organization must be built in monomer-dimer-tetramer-octamer like multiples [10^••^,^32^]. Although a class B GPCR, studies on the secretin receptor identified a key role for transmembrane domain IV and here mutation of a pair of amino acids in the middle of this domain was originally suggested to convert the receptor from strict dimer to monomer. This is associated with altered capacity of the agonist secretin to stimulate levels of cyclic AMP. Recent SpIDA-based studies have supported the idea that these specific mutations do substantially reduce the propensity of this receptor to form a dimeric complex but added a degree of further subtlety in suggesting that these mutants modulate the propensity for rather than define a strict monomer to dimer transition [33]. Of course there is no inherent requirement that direct protein-protein interactions define dimeric or oligomeric Class A GPCR organization. Recent cryo-electron microscopy experiments performed on purified 5-HT_2A_ receptor in the presence or absence of molecules of cholesterol were interpreted to show that this receptor was able to form dimers in the presence of cholesterol while, in contrast, it existed only as a monomer when cholesterol was removed [34^•^]. The importance of molecules of cholesterol in determining the oligomerization state of GPCRs has also been highlighted in studies using photo-bleaching image correlation spectroscopy on the serotonin 5-HT_1A_ receptor [35]. Whilst a trimeric population of the 5-HT_1A_ receptor was prevalent in normal cell membranes, depletion of cholesterol appeared to favor the dimeric state of this receptor [35]. As addressed directly by [10^••^], others have also suggested that the lipid composition of plasma membrane could also influence the stability and the mechanism of formation of the quaternary structures of GPCRs [36] and that different cell types might produce different outcomes based on this feature. Moreover, combined modelling and mutagenesis studies were used to predict-specific roles of molecules of cholesterol in stabilizing a tetrameric configuration of the D_3_R [22^••^] whilst computational studies also predict roles of cholesterol in dimerization of a broad range of chemokine receptors [37].

## Conclusions

Many Class A GPCRs are able to form dimers and oligomers. Although this may be transient in many situations, at physiological expression levels dimer and oligomers may represent a substantial population. Growing data indicate the extent and kinetics of such quaternary complexes are regulated by ligand binding and this may have marked significance for the action of therapeutic medicines.

## Conflict of interest statement

Nothing declared.

## References and recommended reading

Papers of particular interest, published within the period of review, have been highlighted as:

• of special interest
•• of outstanding interest
